# 2,5-Dimethyl-3-phenyl­sulfonyl-1-benzofuran

**DOI:** 10.1107/S1600536808009811

**Published:** 2008-04-16

**Authors:** Hong Dae Choi, Pil Ja Seo, Byeng Wha Son, Uk Lee

**Affiliations:** a Department of Chemistry, Dongeui University, San 24 Kaya-dong, Busanjin-gu, Busan 614-714, Republic of Korea; b Department of Chemistry, Pukyong National University, 599-1 Daeyeon 3-dong, Nam-gu, Busan 608-737, Republic of Korea

## Abstract

The title compound, C_16_H_14_O_3_S, was prepared by the oxidation of 2,5-dimethyl-3-phenyl­sulfanyl-1-benzofuran with 3-chloro­peroxy­benzoic acid. The phenyl ring makes a dihedral angle of 76.98 (9)° with the plane of the benzofuran fragment. The crystal structure is stabilized by π–π inter­actions between furan and benzene rings of neighbouring mol­ecules [centroid–centroid distance = 3.775 (4) Å]. In addition, the crystal structure exhibits intra- and inter­molecular C—H⋯O inter­actions.

## Related literature

For the crystal structures of similar 3-phenyl­sulfonyl-1-benzo­furan derivatives, see: Choi *et al.* (2008*a* ▶,*b* ▶).
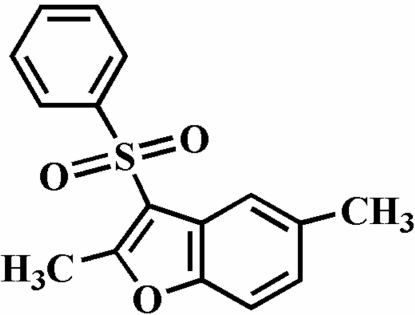


## Experimental

### 

#### Crystal data

C_16_H_14_O_3_S
*M* *_r_* = 286.33
Triclinic, 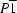

*a* = 7.476 (4) Å
*b* = 9.448 (5) Å
*c* = 11.283 (6) Å
α = 110.834 (8)°
β = 95.651 (9)°
γ = 106.122 (9)°
*V* = 698.0 (6) Å^3^
*Z* = 2
Mo *K*α radiation
μ = 0.24 mm^−1^
*T* = 298 (2) K
0.40 × 0.40 × 0.20 mm

#### Data collection

Bruker SMART CCD diffractometer
Absorption correction: none
4916 measured reflections
2560 independent reflections
1967 reflections with *I* > 2σ(*I*)
*R*_int_ = 0.077

#### Refinement

*R*[*F* ^2^ > 2σ(*F* ^2^)] = 0.057
*wR*(*F* ^2^) = 0.165
*S* = 1.08
2560 reflections
181 parameters
H-atom parameters constrained
Δρ_max_ = 0.33 e Å^−3^
Δρ_min_ = −0.33 e Å^−3^

### 

Data collection: *SMART* (Bruker, 2001 ▶); cell refinement: *SAINT* (Bruker, 2001 ▶); data reduction: *SAINT*; program(s) used to solve structure: *SHELXS97* (Sheldrick, 2008 ▶); program(s) used to refine structure: *SHELXL97* (Sheldrick, 2008 ▶); molecular graphics: *ORTEP-3* (Farrugia, 1997 ▶) and *DIAMOND* (Brandenburg, 1998 ▶); software used to prepare material for publication: *SHELXL97*.

## Supplementary Material

Crystal structure: contains datablocks global, I. DOI: 10.1107/S1600536808009811/sj2482sup1.cif

Structure factors: contains datablocks I. DOI: 10.1107/S1600536808009811/sj2482Isup2.hkl

Additional supplementary materials:  crystallographic information; 3D view; checkCIF report

## Figures and Tables

**Table 1:** Hydrogen-bond geometry (Å, °)

*D*—H⋯*A*	*D*—H	H⋯*A*	*D*⋯*A*	*D*—H⋯*A*
C10—H10⋯O2^i^	0.93	2.45	3.345 (4)	160
C14—H14⋯O3^ii^	0.93	2.50	3.263 (4)	139
C16—H16*C*⋯O3	0.96	2.40	3.108 (4)	130

Symmetry codes: (i) 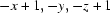; (ii) 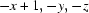.

**Fig. 1.**
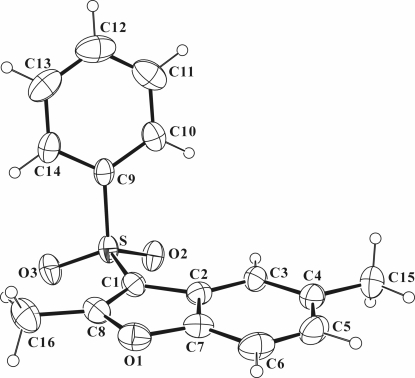
The molecular structure of the title compound, showing displacement ellipsoids drawn at the 30% probability level.

**Fig. 2.**
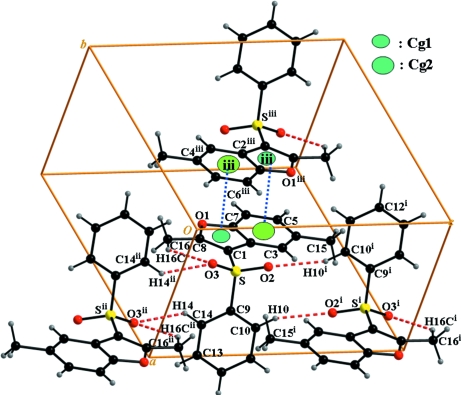
π—π and C—H···O interactions (dotted lines) in the title compound. Cg denotes the ring centroids. [Symmetry code: (i)-x + 1, -y, -z + 1: (ii) -x + 1, -y, -z, (iii) -x + 1, -y + 1, -z + 1.]

## supplementary crystallographic information

### Comment

As a part of our ongoing studies on the synthesis and structure of 3-phenyl-sulfonyl-1-benzofuran analogues, the crystal structure of 5-bromo-2-methyl-3-phenylsulfonyl-1-benzofuran (Choi *et al.*, 2008*a*) and 2,5,7-trimethyl-3-phenylsulfonyl-1-benzofuran (Choi *et al.*, 2008*b*) have been described in the literature. Herein we report the molecular and crystal structure of the title compound, 2,5-dimethyl-3-phenylsulfonyl-1-benzofuran (Fig. 1).

The benzofuran unit is essentially planar, with a mean deviation of 0.065 Å from the least-squares plane defined by the nine constituent atoms. The phenyl ring (C9—C14) makes a dihedral angle of 76.98 (9)° with the plane of the benzofuran fragment. The crystal packing (Fig. 2) is stabilized by aromatic π—π stacking interactions between the furan ring and the benzene ring from neighbouring molecules. The *Cg*1···*Cg*2^iii^ distance is 3.775 (4) Å (*Cg*1 and *Cg*2 are the centroids of the O1/C8/C1/C2/C7 furan and the C2—C7 benzene rings, respectively, symmetry code as in Fig. 2). The molecular packing (Fig. 2) is further stabilized by intra- and intermolecular C—H···O interactions (Table 1 and Fig. 2; symmetry codes as in Fig. 2).

### Experimental

3-Chloroperoxybenzoic acid (77%, 717 mg, 3.2 mmol) was added in small portions to a stirred solution of 2,5-dimethyl 3-phenylsulfanyl-1-benzofuran (381 mg, 1.5 mmol) in dichloromethane (30 ml) at 273 K. After being stirred for 4 h at room temperature, the mixture was washed with saturated sodium bicarbonate solution and the organic layer was separated, dried over magnesium sulfate, filtered and concentrated in vacuum. The residue was purified by column chromatography (hexane-ethyl acetate, 2: 1 *v*/*v*) to afford the title compound as a colorless solid [yield 83%, m.p. 411–412 K; *R*_f_ = 0.67 (hexane-ethyl acetate, 2: 1 *v*/*v*)]. Single crystals suitable for X-ray diffraction were prepared by evaporation of a solution of the title compound in benzene at room temperature. Spectroscopic analysis: 1H NMR (CDCl_3_, 400 MHz) δ 2.45 (s, 3H), 2.79 (s, 3H), 7.10 (d, J = 8.44 Hz, 1H), 7.29 (d, J = 8.44 Hz, 1H), 7.47–7.60 (m, 3H), 7.67 (s, 1H), 7.97–8.03 (m, 2H); EI—MS 286 [*M*^+^].

### Refinement

All H atoms were positioned geometrically and refined using a riding model, with C—H = 0.93 Å for aromatic H atoms and 0.96 Å for methyl H atoms, respectively, and with *U*_iso_(H) = 1.2Ueq(C) for aromatic and 1.5Ueq(C) for methyl H atoms.

### Crystal data

**Table:**

C_16_H_14_O_3_S	*Z* = 2
*M**_r_* = 286.33	*F*_000_ = 300
Triclinic, *P*1	*D*_x_ = 1.362 Mg m^−^^3^
Hall symbol: -P 1	Mo *K*α radiation λ = 0.71073 Å
*a* = 7.476 (4) Å	Cell parameters from 2576 reflections
*b* = 9.448 (5) Å	θ = 2.5–28.2º
*c* = 11.283 (6) Å	µ = 0.24 mm^−^^1^
α = 110.834 (8)º	*T* = 298 (2) K
β = 95.651 (9)º	Block, colorless
γ = 106.122 (9)º	0.40 × 0.40 × 0.20 mm
*V* = 698.0 (6) Å^3^	

### Data collection

**Table:**

Bruker SMART CCD diffractometer	2560 independent reflections
Radiation source: fine-focus sealed tube	1967 reflections with *I* > 2σ(*I*)
Monochromator: graphite	*R*_int_ = 0.077
Detector resolution: 10.0 pixels mm^-1^	θ_max_ = 25.5º
*T* = 298(2) K	θ_min_ = 2.0º
φ and ω scans	*h* = −9→9
Absorption correction: none	*k* = −11→11
4916 measured reflections	*l* = −13→13

### Refinement

**Table:**

Refinement on *F*^2^	Secondary atom site location: difference Fourier map
Least-squares matrix: full	Hydrogen site location: inferred from neighbouring sites
*R*[*F*^2^ > 2σ(*F*^2^)] = 0.057	H-atom parameters constrained
*wR*(*F*^2^) = 0.165	*w* = 1/[σ^2^(*F*_o_^2^) + (0.0708*P*)^2^ + 0.3092*P*] where *P* = (*F*_o_^2^ + 2*F*_c_^2^)/3
*S* = 1.08	(Δ/σ)_max_ < 0.001
2560 reflections	Δρ_max_ = 0.33 e Å^−^^3^
181 parameters	Δρ_min_ = −0.32 e Å^−^^3^
Primary atom site location: structure-invariant direct methods	Extinction correction: none

### Special details

**Table:**

Geometry. All e.s.d.'s (except the e.s.d. in the dihedral angle between two l.s. planes) are estimated using the full covariance matrix. The cell e.s.d.'s are taken into account individually in the estimation of e.s.d.'s in distances, angles and torsion angles; correlations between e.s.d.'s in cell parameters are only used when they are defined by crystal symmetry. An approximate (isotropic) treatment of cell e.s.d.'s is used for estimating e.s.d.'s involving l.s. planes.
Refinement. Refinement of *F*^2^ against ALL reflections. The weighted *R*-factor *wR* and goodness of fit *S* are based on *F*^2^, conventional *R*-factors *R* are based on *F*, with *F* set to zero for negative *F*^2^. The threshold expression of *F*^2^ > σ(*F*^2^) is used only for calculating *R*-factors(gt) *etc*. and is not relevant to the choice of reflections for refinement. *R*-factors based on *F*^2^ are statistically about twice as large as those based on *F*, and *R*- factors based on ALL data will be even larger.

### Fractional atomic coordinates and isotropic or equivalent isotropic displacement parameters (Å^2^)

**Table:**

	*x*	*y*	*z*	*U*_iso_*/*U*_eq_	
S	0.48253 (10)	0.09684 (9)	0.28377 (7)	0.0479 (3)	
O1	0.7729 (3)	0.5526 (2)	0.3996 (2)	0.0621 (6)	
O2	0.3828 (3)	0.0486 (3)	0.37248 (19)	0.0587 (6)	
O3	0.3765 (3)	0.0689 (3)	0.1608 (2)	0.0660 (6)	
C1	0.6127 (4)	0.2991 (3)	0.3636 (3)	0.0469 (7)	
C2	0.6846 (4)	0.3858 (3)	0.5024 (3)	0.0457 (7)	
C3	0.6797 (4)	0.3508 (3)	0.6115 (3)	0.0492 (7)	
H3	0.6177	0.2466	0.6032	0.059*	
C4	0.7678 (5)	0.4719 (4)	0.7328 (3)	0.0585 (8)	
C5	0.8626 (5)	0.6271 (4)	0.7433 (4)	0.0672 (9)	
H5	0.9217	0.7078	0.8254	0.081*	
C6	0.8716 (5)	0.6647 (4)	0.6369 (4)	0.0679 (10)	
H6	0.9349	0.7686	0.6452	0.082*	
C7	0.7827 (4)	0.5416 (3)	0.5174 (3)	0.0523 (8)	
C8	0.6696 (4)	0.4052 (4)	0.3078 (3)	0.0551 (8)	
C9	0.6558 (4)	0.0025 (3)	0.2563 (3)	0.0453 (7)	
C10	0.7288 (5)	−0.0414 (4)	0.3494 (3)	0.0573 (8)	
H10	0.6869	−0.0227	0.4266	0.069*	
C11	0.8658 (6)	−0.1138 (4)	0.3250 (5)	0.0807 (12)	
H11	0.9164	−0.1452	0.3862	0.097*	
C12	0.9277 (6)	−0.1397 (4)	0.2115 (5)	0.0897 (14)	
H12	1.0212	−0.1875	0.1965	0.108*	
C13	0.8531 (6)	−0.0959 (5)	0.1201 (4)	0.0846 (13)	
H13	0.8956	−0.1144	0.0430	0.102*	
C14	0.7170 (5)	−0.0252 (4)	0.1413 (3)	0.0609 (9)	
H14	0.6658	0.0042	0.0789	0.073*	
C15	0.7617 (6)	0.4376 (5)	0.8531 (3)	0.0803 (11)	
H15A	0.8192	0.3571	0.8480	0.096*	
H15B	0.6315	0.3995	0.8596	0.096*	
H15C	0.8307	0.5343	0.9283	0.096*	
C16	0.6474 (6)	0.3918 (5)	0.1724 (4)	0.0784 (11)	
H16A	0.7708	0.4175	0.1517	0.094*	
H16B	0.5864	0.4652	0.1627	0.094*	
H16C	0.5708	0.2839	0.1146	0.094*	

### Atomic displacement parameters (Å^2^)

**Table:**

	*U*^11^	*U*^22^	*U*^33^	*U*^12^	*U*^13^	*U*^23^
S	0.0448 (4)	0.0527 (4)	0.0352 (4)	0.0039 (3)	0.0014 (3)	0.0167 (3)
O1	0.0632 (13)	0.0498 (12)	0.0805 (17)	0.0183 (10)	0.0197 (12)	0.0338 (12)
O2	0.0548 (12)	0.0617 (13)	0.0447 (13)	0.0014 (10)	0.0117 (10)	0.0183 (10)
O3	0.0587 (12)	0.0829 (16)	0.0470 (14)	0.0146 (12)	−0.0054 (11)	0.0268 (12)
C1	0.0428 (14)	0.0491 (16)	0.0488 (18)	0.0137 (12)	0.0075 (13)	0.0217 (14)
C2	0.0395 (13)	0.0424 (15)	0.0505 (18)	0.0135 (11)	0.0072 (13)	0.0141 (13)
C3	0.0510 (15)	0.0442 (15)	0.0435 (18)	0.0126 (13)	0.0034 (14)	0.0118 (13)
C4	0.0577 (17)	0.0580 (19)	0.050 (2)	0.0242 (15)	0.0027 (15)	0.0097 (15)
C5	0.0649 (19)	0.0506 (18)	0.060 (2)	0.0172 (16)	−0.0003 (17)	−0.0023 (16)
C6	0.0588 (19)	0.0379 (16)	0.089 (3)	0.0111 (14)	0.0075 (19)	0.0111 (17)
C7	0.0473 (15)	0.0450 (16)	0.066 (2)	0.0183 (13)	0.0126 (15)	0.0221 (15)
C8	0.0525 (17)	0.0607 (19)	0.061 (2)	0.0214 (15)	0.0129 (15)	0.0324 (17)
C9	0.0481 (14)	0.0387 (13)	0.0333 (15)	0.0015 (11)	−0.0006 (12)	0.0089 (11)
C10	0.0599 (18)	0.0500 (17)	0.0522 (19)	0.0051 (15)	−0.0004 (15)	0.0228 (15)
C11	0.075 (2)	0.052 (2)	0.103 (3)	0.0119 (18)	−0.013 (2)	0.032 (2)
C12	0.074 (2)	0.051 (2)	0.121 (4)	0.0243 (19)	0.013 (3)	0.007 (2)
C13	0.092 (3)	0.064 (2)	0.076 (3)	0.023 (2)	0.030 (2)	0.002 (2)
C14	0.072 (2)	0.0576 (19)	0.0381 (17)	0.0135 (16)	0.0081 (16)	0.0098 (14)
C15	0.098 (3)	0.080 (3)	0.047 (2)	0.034 (2)	−0.003 (2)	0.0103 (18)
C16	0.087 (3)	0.089 (3)	0.078 (3)	0.028 (2)	0.020 (2)	0.056 (2)

### Geometric parameters (Å, °)

**Table:**

S—O3	1.425 (2)	C8—C16	1.476 (4)
S—O2	1.431 (2)	C9—C10	1.376 (4)
S—C1	1.726 (3)	C9—C14	1.380 (4)
S—C9	1.758 (3)	C10—C11	1.380 (5)
O1—C8	1.353 (4)	C10—H10	0.9300
O1—C7	1.366 (4)	C11—C12	1.367 (6)
C1—C8	1.359 (4)	C11—H11	0.9300
C1—C2	1.446 (4)	C12—C13	1.366 (6)
C2—C3	1.385 (4)	C12—H12	0.9300
C2—C7	1.389 (4)	C13—C14	1.360 (6)
C3—C4	1.381 (4)	C13—H13	0.9300
C3—H3	0.9300	C14—H14	0.9300
C4—C5	1.396 (5)	C15—H15A	0.9600
C4—C15	1.505 (5)	C15—H15B	0.9600
C5—C6	1.370 (5)	C15—H15C	0.9600
C5—H5	0.9300	C16—H16A	0.9600
C6—C7	1.375 (5)	C16—H16B	0.9600
C6—H6	0.9300	C16—H16C	0.9600
			
O3—S—O2	118.96 (14)	C10—C9—C14	121.2 (3)
O3—S—C1	109.04 (14)	C10—C9—S	119.9 (2)
O2—S—C1	107.44 (13)	C14—C9—S	119.0 (3)
O3—S—C9	108.12 (14)	C9—C10—C11	118.3 (3)
O2—S—C9	107.90 (14)	C9—C10—H10	120.9
C1—S—C9	104.45 (14)	C11—C10—H10	120.9
C8—O1—C7	107.4 (2)	C12—C11—C10	120.5 (4)
C8—C1—C2	107.5 (3)	C12—C11—H11	119.8
C8—C1—S	126.5 (3)	C10—C11—H11	119.8
C2—C1—S	126.0 (2)	C13—C12—C11	120.4 (4)
C3—C2—C7	119.1 (3)	C13—C12—H12	119.8
C3—C2—C1	136.9 (3)	C11—C12—H12	119.8
C7—C2—C1	104.0 (3)	C14—C13—C12	120.3 (4)
C4—C3—C2	119.4 (3)	C14—C13—H13	119.8
C4—C3—H3	120.3	C12—C13—H13	119.8
C2—C3—H3	120.3	C13—C14—C9	119.4 (4)
C3—C4—C5	119.4 (3)	C13—C14—H14	120.3
C3—C4—C15	120.5 (3)	C9—C14—H14	120.3
C5—C4—C15	120.1 (3)	C4—C15—H15A	109.5
C6—C5—C4	122.4 (3)	C4—C15—H15B	109.5
C6—C5—H5	118.8	H15A—C15—H15B	109.5
C4—C5—H5	118.8	C4—C15—H15C	109.5
C5—C6—C7	116.9 (3)	H15A—C15—H15C	109.5
C5—C6—H6	121.6	H15B—C15—H15C	109.5
C7—C6—H6	121.6	C8—C16—H16A	109.5
O1—C7—C6	126.5 (3)	C8—C16—H16B	109.5
O1—C7—C2	110.7 (3)	H16A—C16—H16B	109.5
C6—C7—C2	122.8 (3)	C8—C16—H16C	109.5
O1—C8—C1	110.4 (3)	H16A—C16—H16C	109.5
O1—C8—C16	115.5 (3)	H16B—C16—H16C	109.5
C1—C8—C16	134.1 (3)		
			
O3—S—C1—C8	−24.0 (3)	C3—C2—C7—C6	−1.8 (5)
O2—S—C1—C8	−154.2 (3)	C1—C2—C7—C6	178.9 (3)
C9—S—C1—C8	91.3 (3)	C7—O1—C8—C1	0.0 (3)
O3—S—C1—C2	157.4 (2)	C7—O1—C8—C16	−178.4 (3)
O2—S—C1—C2	27.2 (3)	C2—C1—C8—O1	0.3 (4)
C9—S—C1—C2	−87.2 (3)	S—C1—C8—O1	−178.5 (2)
C8—C1—C2—C3	−179.4 (3)	C2—C1—C8—C16	178.2 (4)
S—C1—C2—C3	−0.6 (5)	S—C1—C8—C16	−0.6 (6)
C8—C1—C2—C7	−0.4 (3)	O3—S—C9—C10	−156.0 (2)
S—C1—C2—C7	178.4 (2)	O2—S—C9—C10	−26.2 (2)
C7—C2—C3—C4	1.7 (4)	C1—S—C9—C10	87.9 (2)
C1—C2—C3—C4	−179.4 (3)	O3—S—C9—C14	24.4 (3)
C2—C3—C4—C5	−0.9 (5)	O2—S—C9—C14	154.3 (2)
C2—C3—C4—C15	179.0 (3)	C1—S—C9—C14	−91.6 (2)
C3—C4—C5—C6	0.2 (6)	C14—C9—C10—C11	0.2 (4)
C15—C4—C5—C6	−179.8 (3)	S—C9—C10—C11	−179.4 (2)
C4—C5—C6—C7	−0.2 (5)	C9—C10—C11—C12	0.5 (5)
C8—O1—C7—C6	−178.7 (3)	C10—C11—C12—C13	−0.8 (6)
C8—O1—C7—C2	−0.2 (3)	C11—C12—C13—C14	0.3 (6)
C5—C6—C7—O1	179.4 (3)	C12—C13—C14—C9	0.3 (5)
C5—C6—C7—C2	1.1 (5)	C10—C9—C14—C13	−0.6 (4)
C3—C2—C7—O1	179.6 (2)	S—C9—C14—C13	179.0 (2)
C1—C2—C7—O1	0.4 (3)		

### Hydrogen-bond geometry (Å, °)

**Table:**

*D*—H···*A*	*D*—H	H···*A*	*D*···*A*	*D*—H···*A*
C10—H10···O2^i^	0.93	2.45	3.345 (4)	160
C14—H14···O3^ii^	0.93	2.50	3.263 (4)	139
C16—H16C···O3	0.96	2.40	3.108 (4)	130

Symmetry codes: (i) −*x*+1, −*y*, −*z*+1; (ii) −*x*+1, −*y*, −*z*.
